# Probiotic *Bacillus licheniformis* ZW3 Alleviates DSS-Induced Colitis and Enhances Gut Homeostasis

**DOI:** 10.3390/ijms25010561

**Published:** 2024-01-01

**Authors:** Dan Jia, Yingying Li, Yingjie Wang, Yanan Guo, Junlong Liu, Shuaiyang Zhao, Jinming Wang, Guiquan Guan, Jianxun Luo, Hong Yin, Lijie Tang, Youquan Li

**Affiliations:** 1 State Key Laboratory of Veterinary Etiological Biology, Key Laboratory of Veterinary Parasitology of Gansu Province, Lanzhou Veterinary Research Institute, Chinese Academy of Agricultural Sciences, Lanzhou 730046, China; jiadan1995@163.com (D.J.); yingyingli5410@126.com (Y.L.); wangyingjie098@163.com (Y.W.); gyn330@126.com (Y.G.); liujunlong@caas.cn (J.L.); zhaoshuaiyang@caas.cn (S.Z.); wjm0403@caas.cn (J.W.); guanguiquan@caas.cn (G.G.); luojianxun@caas.cn (J.L.); yinhong@caas.cn (H.Y.); 2 College of Veterinary Medicine, Northeast Agricultural University, Harbin 150030, China; 3 College of Coastal Agricultural Sciences, Department of Veterinary Medicine, Guangdong Ocean University, Zhanjiang 524088, China

**Keywords:** *Bacillus licheniformis*, colitis, intestinal mucosal barrier, gut microbiota, purine metabolite, uric acid

## Abstract

Despite *Bacillus* species having been extensively utilized in the food industry and biocontrol as part of probiotic preparations, limited knowledge exists regarding their impact on intestinal disorders. In this study, we investigated the effect of *Bacillus licheniformis* ZW3 (ZW3), a potential probiotic isolated from camel feces, on dextran sulfate sodium (DSS)-induced colitis. The results showed ZW3 partially mitigated body weight loss, disease activity index (DAI), colon shortening, and suppressed immune response in colitis mice, as evidenced by the reduction in the levels of the inflammatory markers IL-1β, TNF-α, and IL-6 (*p* < 0.05). ZW3 was found to ameliorate DSS-induced dysfunction of the colonic barrier by enhancing mucin 2 (MUC2), zonula occluden-1 (ZO-1), and occludin. Furthermore, enriched beneficial bacteria *Lachnospiraceae_NK4A136_group* and decreased harmful bacteria *Escherichia-Shigella* revealed that ZW3 improved the imbalanced gut microbiota. Abnormally elevated uric acid levels in colitis were further normalized upon ZW3 supplementation. Overall, this study emphasized the protective effects of ZW3 in colitis mice as well as some potential applications in the management of inflammation-related diseases.

## 1. Introduction

Ulcerative colitis (UC), a subtype of inflammatory bowel disease (IBD), is a chronic gastrointestinal condition characterized by nonspecific inflammation primarily affecting the colon and rectum. Common clinical symptoms of UC include diarrhea, rectal bleeding, abdominal pain, and the risk of relapse [1]. Despite its global prevalence and substantial economic impact, the pathogenesis of UC remains poorly understood. It has been linked to factors such as heredity, autoimmunity, and alterations in the composition of the gut microbiota [2]. Patients with UC often experience abnormal immune function, dysbiosis of the gut microbiota, and compromised integrity of the intestinal epithelial barrier, leading to increased bacterial access to the mucosal lining and aggravation of intestinal inflammation. Traditional therapeutic approaches for UC mainly involve the use of amino-salicylates, glucocorticoids, and immunosuppressive drugs, which are associated with significant side effects and poor patient compliance [3,4]. In the search for new treatment options, probiotics have emerged as promising biological agents for IBD. Unlike the aforementioned medications, probiotics offer systemic benefits to the host without adverse effects.

Probiotics are defined as live microorganisms that, when administered in adequate amounts, confer health benefits to the host [5]. Research has shown that probiotic supplementation plays a crucial role in optimizing host health and offers a wide range of advantages, including the production of antimicrobial substances, enhancement of mucosal barrier integrity, maintenance of gut flora balance, and modulation of immune responses [6]. The effectiveness of probiotics in disease management has also been demonstrated in various studies [7,8,9]. Probiotic-based drug delivery systems also offer significant advantages in disease treatment [10]. However, most potential probiotics that have demonstrated efficacy against diseases in previous studies belong to the lactic acid bacteria (LAB) group, primarily represented by *Lactobacillus* spp. and *Bifidobacterium* spp. [11]. Surprisingly, there has been relatively limited research on the effects of *Bacillus* species in the prevention or treatment of IBD, and the underlying mechanisms remain unclear.

*Bacillus*, an essential component of probiotics, is a Gram-positive bacterium capable of forming spores. It has gained widespread recognition for its role as a biotechnological producer of enzymes, antibiotics, and bioactive peptides, owing to its efficient secretion capacity and ability to thrive in various fermentation conditions [12]. Additionally, *Bacillus* species have been employed in plant biocontrol and agricultural pest management strategies [13,14]. Notably, the spore-forming nature of *Bacillus* confers high resistance and makes them well-suited for processing, storage, and survival in the gastrointestinal (GI) tract, establishing their potential as probiotics [15]. Our previous research showed an extremely high cure rate (over 95%) against newborn calves’ diarrhea with *Bacillus velezensis* JT3-1, a probiotic isolated from yak feces [16]. Studies on broilers [17] and prawns [18] indicated that *Bacillus* can promote animal growth performance, intestinal inflammation, and oxidative stress by regulating specific gut microbes. As such, specific *Bacillus* is a promising probiotic in the aquaculture industry.

*Bacillus licheniformis* ZW3 (ZW3), a strain isolated from camel feces collected in Ningxia province, China, and preserved in our laboratory, has been demonstrated to have a remarkable tolerance to artificial GI juice and exhibit antibacterial activity against pathogens in vitro. Combined with its secure nature observed in long-term gavage in mice, ZW3 was presumed to be a candidate probiotic. In this study, our objective was to investigate the protective effect of ZW3 in a mouse model of DSS-induced colitis and elucidate its underlying mechanism, thus providing a theoretical foundation for the study and application of *Bacillus* species in colitis treatment.

## 2. Results

### 2.1. B. licheniformis ZW3 Pretreatment Alleviating DSS-Induced Colitis

**Figure 1:**
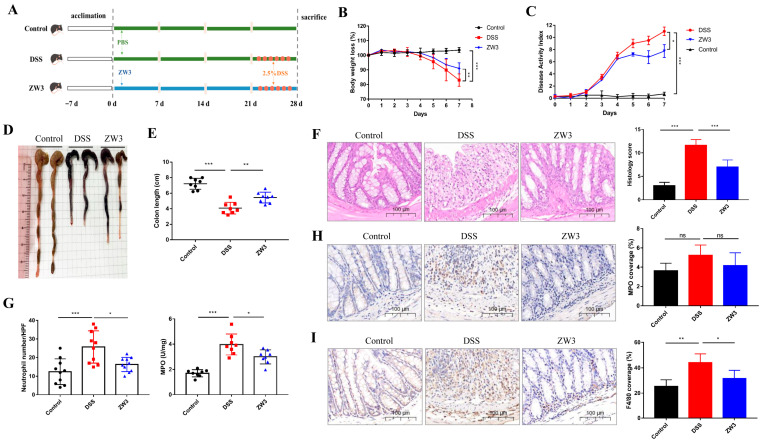
*Bacillus licheniformis* ZW3 (ZW3) attenuates DSS-induced colitis symptoms. (**A**) Schematic of experimental design. (**B**) Body weight loss, (**C**) DIA, (**D**) representative colon images, (**E**) colon length of the mice with free drinking water (control), DSS (DSS), and gavaging ZW3 and DSS (ZW3). (**F**) Representative images of hematoxylin and eosin-stained colonic sections and histological score. (**G**) Neutrophil number/HPF and colonic myeloperoxidase (MPO) activity. Immunohistochemical analysis of the colonic inflammation markers MPO (**H**) and F4/80 (**I**). Scale bar: 100 μm. * *p* < 0.05, ** *p* < 0.01, and *** *p* < 0.001; ns, not significant.

To determine whether ZW3 has a protective effect against DSS-induced colitis, C57BL/6J mice were gavaged with ZW3 for 28 days and accompanied by 2.5% DSS for the last 7 days (Figure 1A). There was no discernible difference in physiological condition among mice in the three groups between days 1 and 21. However, during colitis (21–28 days), mice in the DSS group displayed a significantly greater loss of body weight, a higher DAI score, and a shorter colon compared to mice in the control group, and furthermore, ZW3 treatment considerably remitted these symptoms (*p* < 0.05; Figure 1B–E). We also found that ZW3-treated mice presented a lower colon weight/length ratio and spleen weight than mice in the DSS group (*p* > 0.05; Figure S1). Histopathologic analysis showed administration of ZW3 remedied the luminal impairment caused by DSS, manifested as a reduction in epithelial loss, infiltration of inflammatory cells, and crypt damage. Mice in the control group showed a histological score of 3, whereas mice in the DSS group showed a histological score of 11. However, ZW3 treatment reduced the histological score to nearly 7 in mice that received DSS (Figure 1F).

Furthermore, ZW3 significantly reduced neutrophil number/HPF and colonic myeloperoxidase (MPO) activity in colitis mice (*p* < 0.05; Figure 1G). It was a pity to observe no significant changes in MPO among the three groups via immunohistochemistry assessment (Figure 1H). The DSS-mediated increase in F4/80 coverage rate was significantly reduced upon administration of ZW3 (*p* < 0.05; Figure 1I).

### 2.2. B. licheniformis ZW3 Reduced the Immune Response in DSS-Induced Colitis

**Figure 2:**
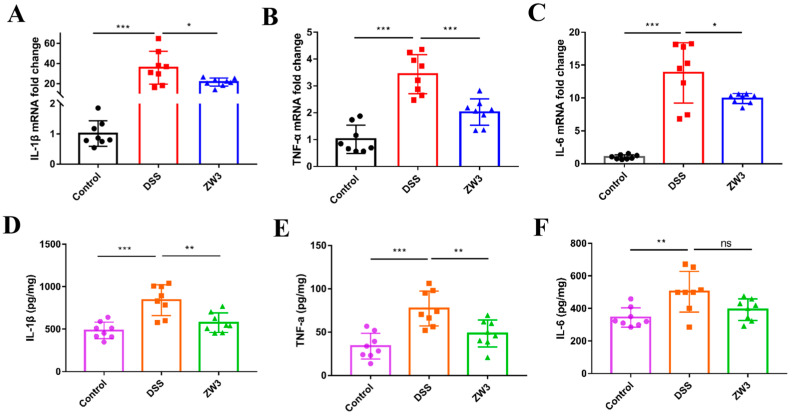
ZW3 suppressed DSS-induced inflammatory responses in colitis mice. Real-time PCR assay for the pro-inflammation genes (**A**) *IL-1β*, (**B**) *TNF-α*, and (**C**) *IL-6* in colonic tissues. ELISA assay for the contents of inflammatory cytokines (**D**) IL-1β, (**E**) TNF-α, and (**F**) IL-6 in the colon tissue. * *p* < 0.05, ** *p* < 0.01, and *** *p* < 0.001; ns, not significant.

We sequentially evaluated proinflammatory markers in colon tissue to better understand the immunomodulatory effects of ZW3 on colitis mice. As shown in Figure 2A–C, qPCR detection confirmed that DSS caused severe intestinal inflammation, as characterized by a remarkable increase in *IL-1β*, *TNF-α*, and *IL-6*. Interestingly, mice responded to ZW3 gavage by showing significantly decreased levels of those cytokines (*p* < 0.05). Consistent with cytokine expression at the transcriptional level, the contents of IL-1β, TNF-α, and IL-6 in colon tissues in the DSS-treated mice were significantly increased compared with those of the control group (*p* < 0.05; Figure 2D–F). As expected, the secretion of IL-1β and TNF-α was significantly decreased in the ZW3 group (*p* < 0.05; Figure 2D,E). The IL-6 level was also slightly lower in ZW3-treated mice than in DSS-treated mice, although this difference was not significant (Figure 2F).

### 2.3. B. licheniformis ZW3 Restored the Intestinal Barrier in DSS-Induced Colitis

**Figure 3:**
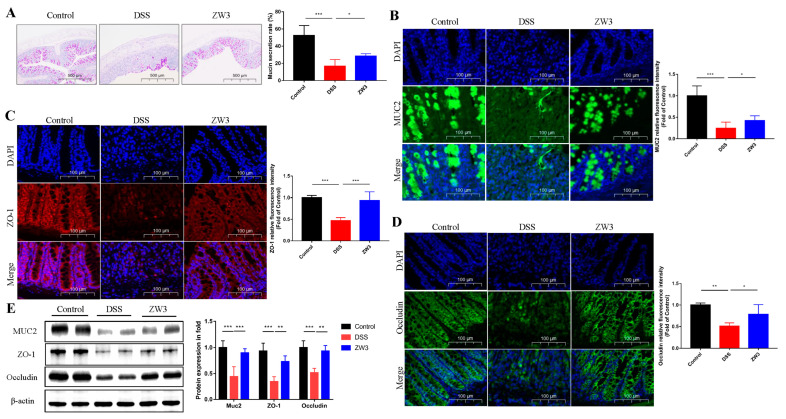
ZW3 attenuated DSS-induced colonic barrier dysfunction. (**A**) PAS staining of the colon with its mucin secretion rate; scale bar: 500 μm. Immunofluorescence staining and its relative fluorescence intensity of MUC2 (**B**), ZO-1 (**C**), and occludin (**D**) in the colonic section from with free drinking water (control), DSS (DSS), and ZW3 plus DSS (ZW3). Blue: DAPI; green: MUC2 and occludin; and red: ZO-1. Scale bar: 100 μm. (**E**) The protein expression and densitometric analysis of MUC2, ZO-1, and occludin. * *p* < 0.05, ** *p* < 0.01, and *** *p* < 0.001.

Goblet cells majorly contribute to the mucus layer and further influence epithelial barrier homeostasis and intestinal permeability. PAS staining showed that DSS treatment resulted in a significant decrease in the number of goblet cells. However, ZW3 administration significantly attenuated these reductions (*p* < 0.05; Figure 3A). Immunofluorescence detection revealed that ZW3 also increased the secretion of MUC2, an essential component of the mucous layer (*p* < 0.05; Figure 3B). In addition, we observed severe loss of ZO-1 and occludin in DSS mice, while ZW3-treated mice had a significant increase in fluorescence intensity (*p* < 0.05; Figure 3C,D). The elevated protein levels of MUC2, ZO-1, and occludin were further confirmed using Western blot (Figure 3E), suggesting that ZW3 could improve the physical structure of the intestinal barrier during colitis.

### 2.4. B. licheniformis ZW3 Restored Intestinal Dysbiosis in Colitis Mice

**Figure 4:**
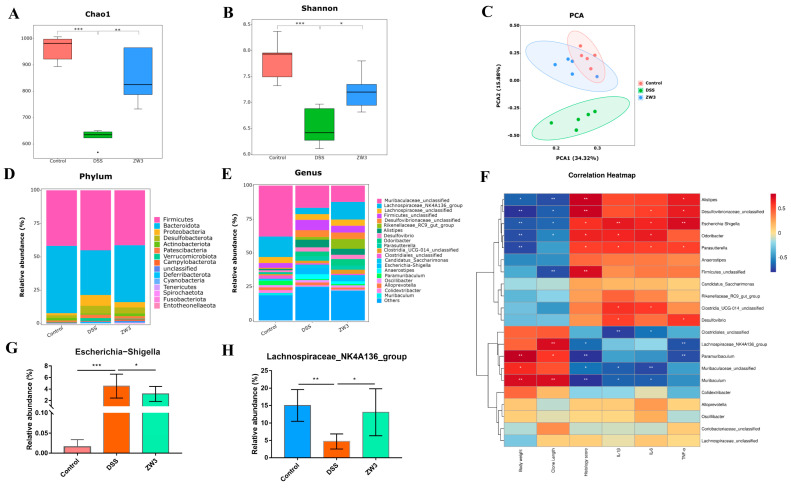
ZW3 regulated the gut microbiota composition in DSS-induced colitis mice. α-diversity of (**A**) Chao1 and (**B**) Shannon index. (**C**) β diversity analysis of the intestinal microbiota using the PCA method. Bar chart of the bacterial community composition at (**D**) the phylum level and (**E**) the genus level. (**F**) Heatmap of the correlation between the relative abundance of bacteria and inflammatory-related mediators using Spearman’s rank correlation test. Relative abundance of (**G**) *Escherichia−Shigella* and (**H**) *Lachnospiraceae_NK4A136_group* at the genus level. Data are shown as the means ± SD. * *p* < 0.05, ** *p* < 0.01, and *** *p* < 0.001 (n = 5).

To determine the contribution of ZW3 to the colon microecological environment in DSS-induced colitis, we performed 16S rRNA sequencing of fecal samples. The α-diversity analysis revealed that the DSS challenge reduced microbial diversity in comparison to the control groups, which could be reversed with ZW3 pretreatment (Figure 4A,B). PCoA analysis exhibited a significant separation of clusters between the DSS and ZW3 groups (Figure 4C), suggesting that ZW3 altered DSS-induced dysbiosis. As shown in Figure 4D, *Firmicutes*, *Bacteroidetes*, and *Proteobacteria* were the predominant flora at the phylum level. DSS treatment caused an obvious decrease in *Bacteroidetes*, while the abundance of *Proteobacteria* increased. However, the administration of ZW3 reverses this pattern. At the genus level, the most abundant were *Muribaculaceae_unclassified* and *Lachnospiraceae_NK4A136_group*, and ZW3 expanded the relative abundance of *Lachnospiraceae_NK4A136_group* compared to the DSS groups (Figure 4E). Spearman’s rank correlation analysis showed that *Alistipes*, *Desulfovibrionaceae_unclassified*, *Escherichia-Shigella*, *Odoribacter*, and *Parasutterella* were positively correlated with the severity of colitis (indicated by body weight, colon length, histology score, IL-1β, IL-6, and TNF-α), whereas bacteria *Clostridiales_unclassified*, *Lachnospiraceae_NK4A136_group*, *Paramuribaculum*, *Muribaculaceae_unclassified*, and *Muribaculum* were negatively correlated (Figure 4F). Interestingly, ZW3 significantly inhibited the abundance of the harmful bacteria *Escherichia-Shigella* and enriched the beneficial bacteria *Lachnospiraceae_NK4A136_group* (*p* < 0.05, Figure 4G,H).

### 2.5. B. licheniformis ZW3 Modulates the Gut Metabolomics Profile in DSS-Induced Colitis

**Figure 5:**
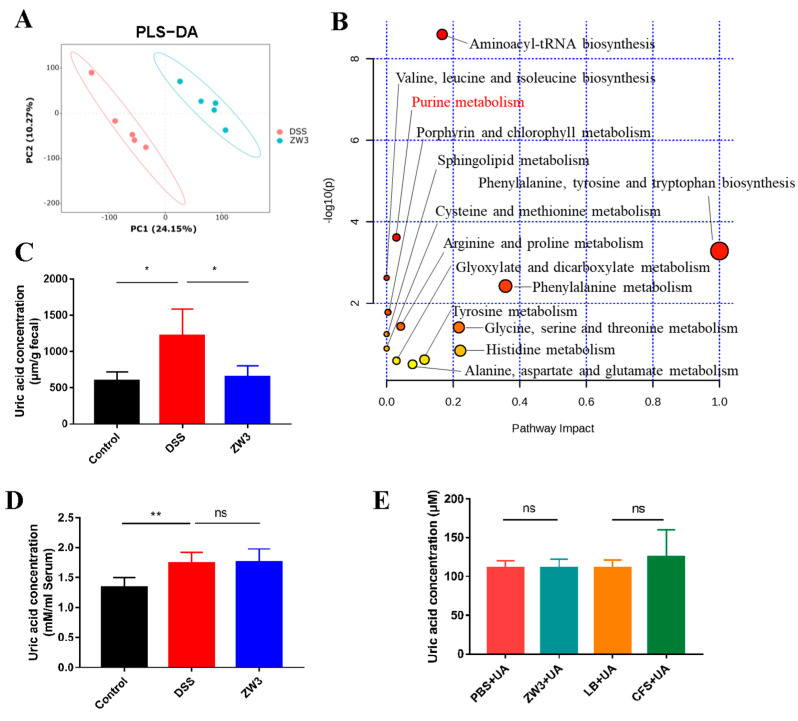
ZW3 altered metabolic profiles in the colitis mice. (**A**) PLS-DA score plot of the DSS and ZW3 groups. (**B**) Pathway enrichment analysis of the significantly altered metabolites in colonic colitis mice and ZW3-treated colitis mice. The color indicated the significance of enrichment, and the redder indicated more significance. Concentrations of uric acid in the feces (**C**) and serum (**D**) of mice (n = 8). (**E**) Effect of ZW3 and its CFS (cell-free supernatant) on uric acid concentration in vitro. Data are means ± SD. * *p* < 0.05, ** *p* < 0.01, and ns, not significant.

We speculated that the protective effect of ZW3 on colitis mice might be related to altered intestinal metabolites. Untargeted metabolomic analysis performed using LC/MS detected 18,045 features in positive mode and 19,045 features in negative mode from mouse colon contents. The partial least squares discrimination analysis (PLS-DA) model exhibited a significant separation of clustering patterns between mice in the DSS group and the ZW3 group (Figure 5A), suggesting that ZW3 effectively regulated the metabolite profile of colitis mice. Based on the strict screening criteria (ratio ≥ 2 or ratio ≤ 1/2, VIP ≥ 1, and *p* value < 0.05) for metabolites with significant differences, a total of 132 metabolites, including 124 upregulated and 8 downregulated metabolites, were identified to be altered between the ZW3- and DSS-treated mice. These metabolites were mainly composed of organic acids and derivatives (54.55%), lipids and lipid-like molecules (15.15%), and organoheterocyclic compounds (10.61%) (Figure S2). The details of key differential metabolites are shown in Table S1. It was determined that cinobufagin, Arg-Leu, Lys-Leu, guanosine, hydroxyprolyl-lysine, and deuteroporphyrin IX were extremely enriched in ZW3-treated mice. However, the levels of uric acid and 2-hydroxyhexanoylglycine decreased significantly.

We then performed pathway enrichment analysis and found that these altered metabolites were mainly enriched in aminoacyl-tRNA biosynthesis; purine metabolism; phenylalanine, tyrosine, and tryptophan biosynthesis; valine, leucine, and isoleucine biosynthesis; and phenylalanine metabolism (Figure 5B). High concentrations of uric acid were detected both in the feces and serum of DSS-treated mice compared with the control group (Figure 5C,D). In contrast, the level of uric acid was significantly decreased in the feces of ZW3-treated mice (*p* < 0.05), whereas there was no significant difference in serum (*p* > 0.05). We further inoculated ZW3 and its CFS (cell-free supernatant) with uric acid in vitro to understand whether ZW3 or its CFS directly degrades uric acid. According to Figure 5E, there was no significant effect of ZW3 and its CFS on uric acid levels.

## 3. Discussion

Despite probiotics being widely known as a health-regulating microbial preparation and accumulated evidence showing that *Lactobacillus* exerts many benefits on the host [19,20], *Bacillus* spp. has received less attention, especially in research on disease control. Here, we conducted the first study of the effects of *Bacillus licheniformis* on DSS-induced colitis mice. Our results confirmed that preventive supplementation with *B. licheniformis* ZW3 could protect mice against colitis, as evidenced by the recovered colon length, body weight, and DAI score. ZW3 also partially improved intestinal structural damage and suppressed the immune response in mice with colitis. The 16S rDNA sequencing and metabolomics analysis revealed that ZW3 ameliorates DSS-induced acute colitis by regulating the gut microbiome and uric acid.

The intestinal epithelial barrier is defined as the first physical line of defense against pathogen invasion [21]. Increased intestinal permeability is a typical marker in patients with IBD [22]. ZO-1, a tight junction protein encoded by the TJP1 gene, has a significant impact on epithelial cell proliferation and mitosis, which is crucial for intestinal mucosal repair [23]. Numerous studies have also shown that ZO-1 is downregulated in IBD. In this study, DSS-treated mice showed a damaged intestinal barrier characterized by decreased expression of ZO-1 and occludin. However, an increased relative fluorescence intensity and protein expression of ZO-1 and occludin were observed in ZW3-treated mice, which is in accordance with previous studies [24]. Moreover, ZW3 treatment significantly increased the content of mucin MUC2, which is considered the main component of the mucus barrier and exerts a protective role in the host intestinal tract.

Apart from the intestinal epithelial barrier, the immune barrier also plays a vital role in the development of colitis, typically accompanied by increased pro-inflammatory cytokines [25,26]. Clinically, many immunosuppressive-based drugs have been developed and applied to treat IBD, such as azathioprine, cyclosporin, and methotrexate [27]. ZW3-treated mice showed less severe inflammation than DSS mice, as evidenced by decreased TNF-α, IL-1β, and IL-6 (Figure 2A–F). Elevated inflammatory cytokines in colitis mice are produced by activated immune cells, and they can further facilitate the activation of inflamed immune cells and cause immune imbalance [28]. In addition, upregulated TNF-α in colitis leads to massive apoptosis and shedding of epithelial cells, thus altering intestinal permeability and ultimately aggravating inflammation [29,30]. IL-1β was also proven to change tight junctions [31]. Surprisingly, administration of ZW3 resulted in a 50% and 40% reduction in TNF-α and IL-1β at the transcriptional level, respectively, compared with the DSS group. These findings demonstrated that ZW3 alleviated DSS-induced colitis by modulating immune homeostasis, which was also confirmed with the decreased proinflammatory macrophage marker F4/80 and neutrophil infiltration marker MPO activity (Figure 1G–I). Dong et al. also found that *Pediococcus pentosaceus* CECT 8330 displayed anti-inflammatory activity by significantly reducing the level of pro-inflammation markers in colitis mice [32].

The health of the host depends largely on intestinal homeostasis. Colitis has been clinically shown to be associated with dysregulated gut microbiota [33,34]. It was reported that inflammatory bowel disease patients often have decreased microbial diversity and increased pathogenic bacteria in the intestinal lumen [35]. Patients with other diseases, such as obesity, gastrointestinal disorders, diabetes, and Alzheimer’s disease, also exhibit dysbiosis of the gut microbiota [36,37]. In the present study, 16S rRNA sequencing showed that DSS treatment resulted in reduced gut microbial richness and diversity in mice, which was effectively recovered with ZW3 pretreatment. As a major fraction of the gut bacteriome, the *Bacteroidetes* phylum is determined to be the only sphingolipid producer among intestinal symbionts, which is essential for maintaining intestinal homeostasis and symbiosis [38,39]. In line with the outcomes of most studies, we observed a poor abundance of *Bacteroidetes* in mice that received DSS and further found that ZW3 pretreatment mitigated the reduction in *Bacteroidetes*. *Muribaculaceae_unclassified* and *Lachnospiraceae_NK4A136_group* were dominant at the genus level in mouse intestines, which was reduced significantly with DSS treatment. A similar change was obtained in the study of Zhang et al. [40]. However, we observed an increased amount of *Lachnospiraceae_NK4A136_group* and *Odoribacter* in the ZW3 group, which were reported to produce short-chain fatty acids (SCFAs), such as propionic, acetic, and butyric acids [41]. In addition, ZW3 significantly reduced the abundance of the pathogen *Escherichia-Shigella* (*p* < 0.05), which was positively correlated with colitis severity. Hence, the protective effect of ZW3 on colitis mice may be achieved by regulating the intestinal flora composition.

One of the primary mechanisms by which the gut microbiota maintains health is through its metabolites [42]. Gut microbiota-derived tryptophan metabolites, such as indole-3-ethanol, indole-3-pyruvate, and indole-3-aldehyde, were proven to regulate gut barrier function and the immune response by activating the aryl hydrocarbon receptor [43,44]. Bile acids (BAs) are potent metabolic and immune signaling molecules, and their composition depends largely on the gut bacterial species [45]. Liu et al. showed that fucoidan improved intestinal barrier function and colonic inflammation by elevating cholic acid, ursodeoxycholic acid, and deoxycholic acid [46]. In this study, untargeted metabolomics of mouse colonic contents was used to understand the modulatory effect of ZW3 on the metabolite profiles of DSS-induced colitis. In contrast to previous findings, the comparative metabolomic analysis revealed that ZW3 significantly altered purine metabolism in colitis mice (Figure 5B). Purines are the most abundant metabolic substrates in living organisms and not only generate DNA and RNA but are also essential for cellular energy and intracellular signaling in cells [47]. Recent studies have revealed that purine metabolism may become a biomarker and therapeutic target for many diseases [48,49,50]. Uric acid is the final metabolite of purine metabolism and is increased in IBD patients [51]. A previous study showed that uric acid administration increased intestinal permeability and reduced mucin content in healthy mice [52]. Another study proved that uric acid plus DSS results in worsened intestinal epithelial damage and colitis compared to DSS treatment alone. Lv et al. proposed that uric acid drives intestinal barrier dysfunction mainly through TSPO-mediated NLRP3 inflammasome activation, suggesting that elevated uric acid may be a culprit in exacerbating colitis [53]. In our study, we confirmed that uric acid was closely related to the development of colitis, and DSS induced a high uric acid level in both mouse serum and feces, which could be indirectly abrogated with ZW3 supplementation. Coincidentally, rhein indirectly modulates host purine metabolism in the intestine through the gut microbiota and ameliorates experimental colitis [52]. Hence, we speculated that ZW3 may also restore intestinal barriers by reducing uric acid levels mediated by the intestinal microbiota in mice with colitis.

## 4. Materials and Methods

### 4.1. Probiotics and Animals

ZW3 was isolated from camel feces collected from Ningxia Province, China, and identified at the species level via 16S ribosomal RNA sequencing (accession number: OR817767). This strain was cultured in Luria Bertani (LB) medium at 37 °C for 24 h aerobically.

A total of 24 six-week-old C57BL/6J male mice were purchased from Lanzhou Veterinary Research Institute, Chinese Academy of Agricultural Sciences, and raised under specific-pathogen-free (SPF) conditions. All mice were kept in a temperature-controlled room (22 ± 2 °C) with a 12 h–12 h dark–light cycle and were provided ad libitum access to food and water.

### 4.2. Experimental Design

After a week of acclimatization, mice were randomly divided into three groups (n = 8): control, DSS, and ZW3 (Figure 1A). Throughout the experiment, the control group and DSS group were intragastrically administered PBS (0.2 mL/mouse/day), while the ZW3 group was gavaged with *B. licheniformis* ZW3 (resuspended in PBS, 2 × 10^8^ CFU/0.2 mL/day). From day 21 to day 28, mice in the control group were provided autoclaved drinking water, while mice in the DSS group and ZW3 group received 2.5% (*w/v*) DSS (MP Biomedicals, Solon, OH, USA) in drinking water to induce acute colitis. During colitis, body weight was recorded, and feces were collected daily for consistency and bloody stool detection. At the end of the experiment, the animals were sacrificed via cervical dislocation, and the entire colon was removed for further study.

### 4.3. DAI

The disease activity index (DAI) was determined by combining body weight loss, bloody stools, and stool consistency. The specific assessment methods were shown in Table S2, and the DAI score was calculated as the sum of the weight loss score, diarrhea score, and rectal bleeding score.

### 4.4. Hematoxylin–Eosin (HE) and Periodic Acid-Schiff (PAS) Staining

A piece of the distal colon was collected and fixed in 4% (*w/v*) paraformaldehyde for 24 h, embedded in paraffin, cut into 5 µm slices, and stained with hematoxylin–eosin (H&E) and periodic acid Schiff reagent (PAS, Servicebio, Wuhan, China). The histopathological score was calculated based on epithelial loss, crypt damage, depletion of goblet cells, and infiltration of inflammatory cells, as shown in Table S3 [54]. The mucin secretion rate was assessed using the number of goblet cells. Neutrophil infiltrating was assessed by manually counting the number of neutrophils in H&E sections in 10 high-power fields (HPF) pictures with a magnification of 400× randomly [55].

### 4.5. Immunohistochemistry (IHC) and Immunofluorescence (IF)

Colon tissue sections with 4 μm thickness were dewaxed, rehydrated, blocked with 5% BSA, and incubated with primary antibodies against MPO and F4/80 overnight at 4 °C. Following washing three times in PBS, slides were further incubated with secondary antibodies and finally visualized via diaminobenzidine (DAB) substrate. Images were acquired via standard microscopy (E100; Nikon, Tokyo, Japan) and a digital camera (Nikon DXm1200F, Nikon, Japan).

For immunofluorescence staining, paraffin-embedded tissues were stained with anti-MUC2 antibody, anti-ZO-1 antibody, and anti-occludin antibody. Then, the sections were successively incubated with secondary antibodies for 1 h and antifade mounting medium (with DAPI) for nuclear counterstaining. Finally, images were captured using fluorescent microscopy (Nikon C1 Eclipse; Nikon, Tokyo, Japan) and an imaging system (Nikon DS-U3; Nikon, Japan), and Image J software (1.8.0, National Institutes of Health, Bethesda, MD, USA) was used to analyze the data. Details of the antibodies are shown in Table S3.

### 4.6. RT-PCR

Total RNA in colonic tissues was extracted using TRIzol reagent (Invitrogen, Carlsbad, CA, USA) and purified via lithium chloride precipitation [56]. cDNA was synthesized from 1 μg of total RNA. Real-time PCR was conducted using TB Green Premix Ex Taq II (TaKaRa, Tokyo, Japan) and performed in the Agilent Mx3005P system (Agilent Technologies, Santa Clara, CA, USA). The transcript levels of each gene were normalized to GAPDH and calculated using the 2^−ΔΔCt^ method. The primers used are listed in Table 1.

### 4.7. Enzyme-Linked Immunosorbent Assays and Myeloperoxidase Activity (MPO)

Samples of the colon were homogenized in ice-cold PBS at a ratio of 1:9 (*w/v*) and centrifuged at 12,000× *g* for 15 min at 4 °C. Inflammation-related cytokines were measured using a TNF-α ELISA kit (Boster, Wuhan, China) and a mouse IL-1β and IL-6 ELISA kit (BioLegend, San Diego, CA, USA). The colonic myeloperoxidase activity was measured using a biochemical kit (Nanjing Jiancheng Bioengineering Institute, Nanjing, China) as recommended by the manufacturer’s instructions.

### 4.8. Western Blot Analysis

Colonic tissue was homogenized in a Tissue Protein Extraction Reagent containing a 1% protease inhibitor cocktail and centrifuged at 12,000× *g* for 15 min to obtain lysates that were quantified with a BCA kit. A total of 30 μg of protein was electrophoresed via SDS-PAGE and then transferred to polyvinylidene fluoride (PVDF) membranes. Membranes were sequentially blocked with 5% skim milk for 1.5 h and incubated with antibodies against MUC2, ZO-1, and occludin at 4 °C overnight. After five 5 min washes in TBST, the membranes were then incubated with secondary antibody-conjugated HRP (1:5000, Abcam) for 1 h at room temperature, and the protein concentration was determined using an ECL kit. The reagents mentioned above were purchased from Thermo Fisher Scientific, Waltham, MA, USA, and the details of the antibodies are shown in Table S4. The results are expressed relative to the housekeeping gene β-actin. Finally, ImageJ software (1.8.0, National Institutes of Health, USA) was used to quantify the protein expression.

### 4.9. 16S rRNA Gene Sequencing

Total bacterial genomic DNA was extracted from feces using the Qiagen DNA isolation kit (Qiagen, Hilden, Germany). The V3-V4 region of bacterial 16S rRNA was amplified using primers 341F (5′-CCTACGGGNGGCWGCAG-3′) and 805R (5′-GACTACHVGGGTATCTAATCC-3′) [57]. Purification and sequencing of the PCR amplification products were performed on the Illumina NovaSeq PE250 sequencing platform (LC-Bio Technology Co., Ltd., Hangzhou, China). After filtration and dereplication of the raw reads, feature sequences were used to calculate alpha diversity and beta diversity with QIIME2 and further annotated with the SILVA database. Other diagrams were implemented using the R package (v3.5.2).

### 4.10. Nontargeted Metabolomics Analysis of Colon Contents

Metabolites from colon contents were extracted with 50% methanol buffer, and the supernatant was transferred for liquid chromatography-mass spectrometry (LC-MS) analysis. All chromatographic separations were performed using a Vanquish Flex UHPLC system (Thermo Fisher Scientific, Bremen, Germany) and an ACQUITY UPLC T3 column (100 mm × 2.1 mm, 1.8 µm, Waters, Milford, MA, USA). The detection of metabolites in eluents was performed using a high-resolution mass spectrometer, Q-Exactive (Thermo Scientific, Waltham, MA, USA), that operated in both positive and negative ion modes.

The raw MS data were converted to mzXML format and then processed using the XCMS, CAMERA, and metaX toolboxes accomplished with R software version 3.4.3 (http://www.R-project.org/, accessed on 25 February 2023). Each ion was identified by combining the retention time and *m*/*z* data. Annotation of metabolites was completed with KEGG and HMDB databases, and an in-house fragment spectrum library of metabolites was also used to validate metabolite identification. LC/MS and untargeted metabolomics raw data were obtained at LC-Bio Technology Co., Ltd., Hangzhou, Zhejiang Province, China.

Student’s *t*-tests were used to detect the differences in metabolite concentrations between the two groups. Partial least squares discriminant analysis (PLS-DA) was performed to establish the relationship between metabolite expression and sample class. The differential metabolites between the two groups were determined by variable importance in the projection (VIP) > 1 from the PLS-DA model and the *p* values (<0.05). The altered metabolites were analyzed using MetaboAnalyst 5.0 (https://www.metaboanalyst.ca/, accessed on 25 February 2023).

### 4.11. Measurement of Uric Acid

For the experiment of ZW3 and its metabolite intervention in uric acid, freshly cultured ZW3 was centrifuged at 8000× *g* for 20 min to collect the cell-free supernatant (CFS), followed by filtration through a 0.22 μm filter (Merck Millipore, Tullagreen, Ireland), while the bacterial particles were resuspended in sterile PBS. The bacterial solution and its CFS were mixed with a sterile uric acid solution (1:9 *v*/*v*) for 12 h at room temperature. Sterile PBS and LB containing uric acid were prepared as controls. The supernatants were collected for further detection.

For the in vivo test, fecal suspensions were prepared by homogenizing the mouse feces in sterile PBS and centrifuged at 10,000× *g* for 10 min. Blood collected through the orbit was left for 2 h at room temperature and then centrifuged at 12,000× *g* for 15 min for serum collection. The uric acid levels in these samples were determined following the instructions provided in the Amplex^®^ Red uric acid/uricase assay kit (Life Technologies, Carlsbad, CA, USA).

### 4.12. Statistical Analyses

All data are presented as the mean ± standard deviation (SD), and the analyses were performed using GraphPad Prism 8.0 software (GraphPad Software, Inc., La Jolla, CA, USA). Significant differences between the two groups were evaluated using a two-tailed, unpaired Student’s *t*-test. Significant differences in the three groups were evaluated using a one-way ANOVA followed by Tukey’s post hoc test. *p* < 0.05 was considered statistically significant.

## 5. Conclusions

In conclusion, the present study indicates that oral administration of ZW3 can partially enhance gut barrier integrity and suppress the inflammatory response in DSS-induced colitis mice. This improvement was closely related to the ZW3-mediated normalization of the gut microbiota and purine metabolism. These results provide a reference and theoretical basis for the application of ZW3 in the prevention of colitis and the development of biologics for the treatment of IBD. It also offers some inspiration for the treatment of other gastrointestinal inflammations. However, the colitis model used in this study is only a chemical model, which has great limitations in predicting clinical utility. Further studies are required to determine how it functions in colitis in humans and animals for a more comprehensive understanding of its potential benefits. In addition, elevated uric acid levels were proposed to be strongly correlated with several diseases, such as gout, diabetes, obesity, and kidney diseases. It is valuable to explore the effect of ZW3 on these diseases.

## Figures and Tables

**Table 1:** List of primers used for qPCR.

Gene	Forward Primer	Reverse Primer
*IL-1β*	TTGACGGACCCCAAAAGAT	AGCTGGATGCTCTCATCAGG
*TNF-α*	GCGACGTGGAACTGGCAGAAG	GCCACAAGCAGGAATGAGAAGAGG
*IL-6*	CCGGAGAGGAGACTTCACAG	CAGAATTGCCATTGCACAAC
*GAPDH*	TGTGTCCGTCGTGGATCTGA	TTGCTGTTGAAGTCGCAGGAG

## Data Availability

The raw data presented in this study can be found in the NCBI repository with accession number PRJNA1028485.

## Supplementary Materials

The following supporting information can be downloaded at https://www.mdpi.com/article/10.3390/ijms25010561/s1.
