# Supplementary material for: Probiotic Bacillus licheniformis ZW3 Alleviates DSS-Induced Colitis and Enhances Gut Homeostasis
Source: Int J Mol Sci. 2024 Jan 1;25(1):561. doi: 10.3390/ijms25010561 (PMC10778761; doi:10.3390/ijms25010561)
Supplement: Supplementary file 1 [file ijms-25-00561-s001.zip › ijms-2765509-supplementary.pdf]

Supplementary Materials

1. Supplementary Figures

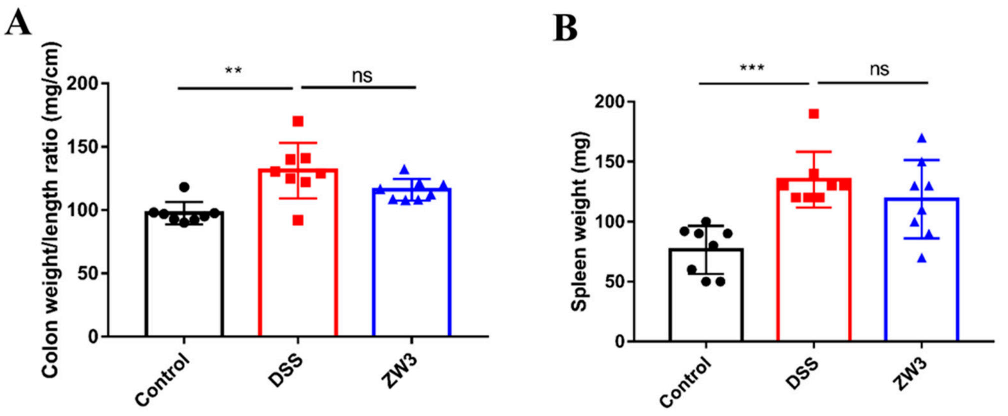

Figure S1. *B. licheniformis* ZW3 attenuates DSS-induced colitis symptoms. (A) Colon weight/length ratio and (B) spleen weight of the mice with free drinking water (Control), DSS (DSS), and ZW3 plus DSS (ZW3). $^{**}p < 0.01$, $^{***}p < 0.001$, and ns, not significant.

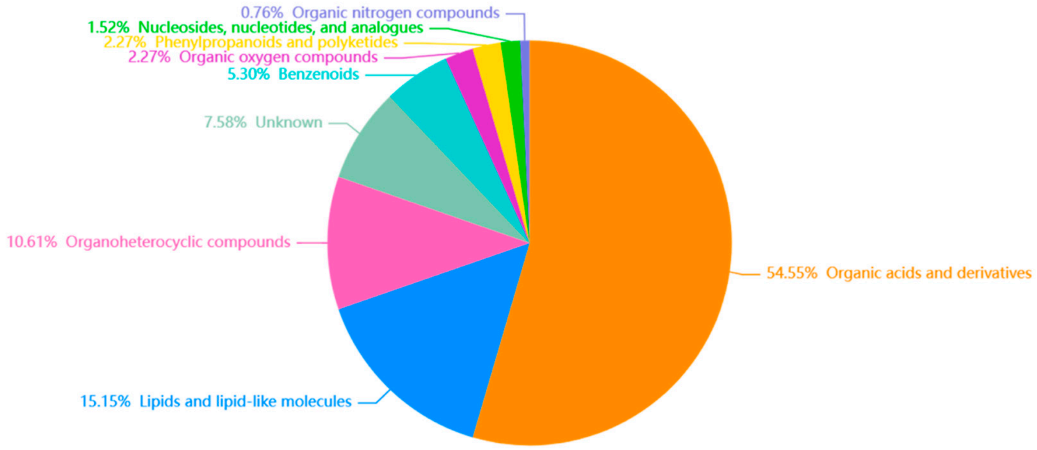

Figure S2. MS2 superclass classification of the differential metabolites between the colitis mice and ZW3-treated colitis mice.

2. Supplementary Tables

Table S1. Metabolomic changes in the colonic contents between mice in the ZW3 group and DSS group

Metabolites	RT (min)	VIP	Ratio	Regulated	Mode
Cinobufagin	4.605	3.92	11.76	↑	-
Arg-Leu	2.260	2.60	7.91	↑	+
Lys-Leu	1.990	2.36	7.57	↑	+
Guanosine	2.801	2.30	6.41	↑	-
Hydroxyprolyl-Lysine	2.249	2.42	6.31	↑	-
Histidinyl-Leucine	1.988	2.54	6.20	↑	+
Deuteroporphyrin IX	4.614	3.30	6.18	↑	-
Guanosine	2.796	2.30	5.97	↑	+
Glutamine	0.940	2.24	4.00	↑	-
Ureidoisobutyric acid	0.978	2.21	3.81	↑	+
Guanine	2.804	1.84	3.49	↑	-
Hypoxanthine	2.797	1.38	2.19	↑	+
Uric acid	1.080	3.64	0.015	↓	-
1-(2-Pyrimidinyl)-4-piperidinamine	8.170	2.21	0.33	↓	+
2-hydroxyhexanoylglycine	3.336	2.08	0.21	↓	-
Stearamide	5.434	1.87	0.36	↓	+
1,4-Diaminonaphthalene	3.510	1.89	0.37	↓	+
3-Oxostearic acid	5.496	1.34	0.44	↓	+

Table S2. Disease activity index (DAI) score of DSS-induced colitis

Score	Weight loss	Bloody stools	Stool consistency
0	None	None	Normal
1	1-5%	-	-
2	6-10%	Blood visible in stool	Loose stools
3	11-15%	-	-
4	Over 15%	Gross bleeding	Diarrhea

Table S3. Histological scores of DSS-induced colitis

Score	Epithelium loss	Crypt loss	Infiltration of inflammatory cells	Depletion of goblet cells
0	None	None	None	None
1	0%-5%	0%-10%	Mild	Mild
2	5%-10%	10%-20%	Medium	Medium
3	Over 10%	Over 20%	Severe	Severe

Table S4. Antibodies used in this study

	Application	Dilution	Manufacturer	Cat. No.	Lot. No.
MPO	IHC	1:1000	Servicebio, Wuhan, China	GB12224	AC2111015A
F4/80	IHC	1:300	Servicebio, Wuhan, China	GB113373	AC210730003
MUC2	IF	1:500	Servicebio, Wuhan, China	GB11344	AC211012453
ZO-1	IF	1:200	Servicebio, Wuhan, China	GB111402	AC211114002
Occludin	IF	1:1000	Servicebio, Wuhan, China	GB111401	AC210814001
MUC2	WB	1:8000	Abcam, Cambridge, MA, USA	Ab272692	1009676-7
ZO-1	WB	1:5000	Proteintech, Wuhan, China	21773-1-AP	00114347
Occludin	WB	1:5000	Proteintech, Wuhan, China	27260-1-AP	00114087
β-actin	WB	1:2000	Servicebio, Wuhan, China	GB15003	AC220918001
